# Impact of Elevated Hemoglobin and Serum Protein on Vasovagal Reaction from Blood Donation

**DOI:** 10.1371/journal.pone.0148854

**Published:** 2016-02-19

**Authors:** Takeshi Odajima, Minoko Takanashi, Hiroki Sugimori, Taiko Tanba, Kentaro Yoshinaga, Toshiko Motoji, Masaya Munakata, Kazunori Nakajima, Mutsuhiko Minami

**Affiliations:** 1 Japanese Red Cross Kanto-Koshinetsu Block Blood Center, Koto-ku, Tokyo, Japan; 2 The Japanese Red Cross Society, Minato-ku, Tokyo, Japan; 3 Department of Sports and Health Science, Daito Bunka University, Higashimatsuyama, Saitama, Japan; 4 Department of Haematology, Tokyo Women's Medical University, Shinjuku-ku, Tokyo, Japan; University Hospital of Salamanca, SPAIN

## Abstract

We conducted a cross-sectional study to elucidate factors contributing to vasovagal reaction (VVR), the most frequent side effect following whole blood and apheresis donations. Complications recorded at the collection sites after voluntary donations by the Japanese Red Cross Tokyo Blood Center (JRC), in the 2006 and 2007 fiscal years, were analyzed by both univariate analysis and the multivariate conditional logistic regression model. Of 1,119,716 blood donations over the full two years, complications were recorded for 13,320 donations (1.18%), among which 67% were VVR. There were 4,303 VVR cases which had sufficient information and could be used for this study. For each VVR case, two sex- and age-matched controls (n = 8,606) were randomly selected from the donors without complications. Age, sex, body mass index (BMI), predonation blood pressure, pulse and blood test results, including total protein, albumin, and hemoglobin, were compared between the VVR group and the control group. In univariate analysis, the VVR group was significantly younger, with a lower BMI, higher blood pressure and higher blood protein and hemoglobin levels than the control group (p<0.001). Furthermore, blood protein and hemoglobin levels showed dose-dependent relationships with VVR incidences by the Cochran-Armitage trend test (p<0.01). For both sexes, after adjusting for confounders with the multivariate conditional logistic regression model, the higher than median groups for total protein (male: OR 1.97; 95%CI 1.76,-2.21; female: OR 2.29; 95%CI 2.05–2.56), albumin (male: 1.75; 1.55–1.96; female: 1.76; 1.57–1.97) and hemoglobin (male: 1.98; 1.76–2.22; female: 1.62; 1.45–1.81) had statistically significant higher risk of VVR compared to the lower than median groups. These elevated serum protein and hemoglobin levels might offer new indicators to help understand VVR occurrence.

## Introduction

The volunteer spirit of healthy donors is the foundation of blood donation. In Japan, blood is collected from approximately 5 million people annually. However, it has been reported that the prevalence of adverse reactions to blood donation is as high as 1%, approximately 75% of which are vasovagal reaction (VVR) [1–3]. Loss of consciousness induced by VVR, vasovagal syncope, may cause unexpected falling, which can result in serious accidents such as tooth damage or even brain damage.

The risk factors for VVR have been studied from various perspectives. There have been reports on the risk from blood donation for first-time donors, youth, females, donors who have a small blood volume, a high pulse rate before blood collection, etc. [4–9]. We have also reported that the VVR risk is associated with a young age, low body mass index (BMI), high blood pressure before blood collection, fast pulse rate, first-time donation, small circulating blood volume (CBV) and short sleep time [10]. Although the risk factors for VVR are not fully elucidated at present, the Japanese Red Cross (JRC) Blood Centers, which are engaged in blood collection, have promoted the prevention of VVR at donation sites and have obtained good results, by distributing posters and brochures to remind blood donors to consume water before and after blood collection and to have sufficient rest after [11].

The relationships between VVR and blood chemical values such as total protein (TP), albumin (Alb) and hemoglobin (Hb) have not been studied sufficiently thus far. We analyzed how these values influence and are related to the occurrence of VVR using the data of blood donors over a 2 year period at the JRC Tokyo Blood Center.

## Materials and Methods

### Blood center procedures

The JRC Blood Centers provide 100% of the blood for transfusion in Japan by collecting whole blood (WB) or apheresis components from volunteer donors. The blood collection includes 200 mL WB (WB200), 400 mL WB (WB400) and apheresis platelet and plasma collection (PC & PPP). An interview by a physician and Hb point-of-care testing is needed before a donation can be made. At the time the donations were made which are used in this study, the donors for WB200 were required to be from 16–69 years old, have a body weight of ≧45kg for males and ≧40kg for females, and an Hb level ≧12.0g/dL. For WB400 donors, their age had to be from 18–69 years old, body weight ≧50 kg and Hb ≧12.5 g/dL. For apheresis, the age had to be 18–54 years old, the body weight ≧45 kg for males and ≧40 kg for females, and the Hb level ≧12 g/dL. These criteria were modified in 2011. As the JRC tries to restrict WB200 collection to first time donors, the WB200 group was not included in this analysis.

We conduct tests for infectious diseases, complete blood counts and biochemical parameters such as alanine aminotransferase, gamma-glutamyl transpeptidase, TP, Alb, total cholesterol and glycated albumin. The JRC sends the data to the donors to help them manage their health as a public health service. All blood donors are allogeneic healthy volunteers. Platelets and plasma were collected using one of the following three blood component-collection systems: Trima (Gambro BCT, Lakewood, CO), TerusysS (Terumo, Tokyo, Japan) or Hemonetics CCS (Hemonetics, Braintree, MA). The CBV (L) is calculated according to Ogawa et al. [12]:

CBV = 0.168H^3^ + 0.050W + 0.444 for an adult male, and

CBV = 0.250H^3^ + 0.0625W - 0.662 for an adult female, where H is the height (m) and W is the weight (kg). The plasma volume to be collected must be ≦12% of the CBV and between 300 and 600mL. For PC apheresis the volume for a bag of ≧2x 10^11 platelets is adjusted to be about 200mL, and with simultaneous plasma collection the total volume must be under 400mL. When any adverse reaction due to blood collection occurs at the collection site, staff members, including physicians, provide treatment on-site. If the reaction is more serious, the donor will be transported to a hospital. All blood donors are instructed to contact the JRC Blood Center if they feel uneasy or have health problems after the donation. The JRC Blood Centers record events that occur during or after blood donation, including outside the donation site.

### VVR group

The adverse reactions of the donors are recorded in the central database system. When a donor presents symptoms including weakness, pallor, yawning, cold sweat, nausea, vomiting, fainting, convulsion or incontinence, with lower systolic blood pressure or a lower pulse rate compared to those recorded at reception, the nurse, consulting with the on-site physician, takes care of the donor. The cases diagnosed as VVR are registered with detailed information related to the blood donation and any physical or psychological stress. The procedure is standardized and a preset form is used.

**Fig 1:**
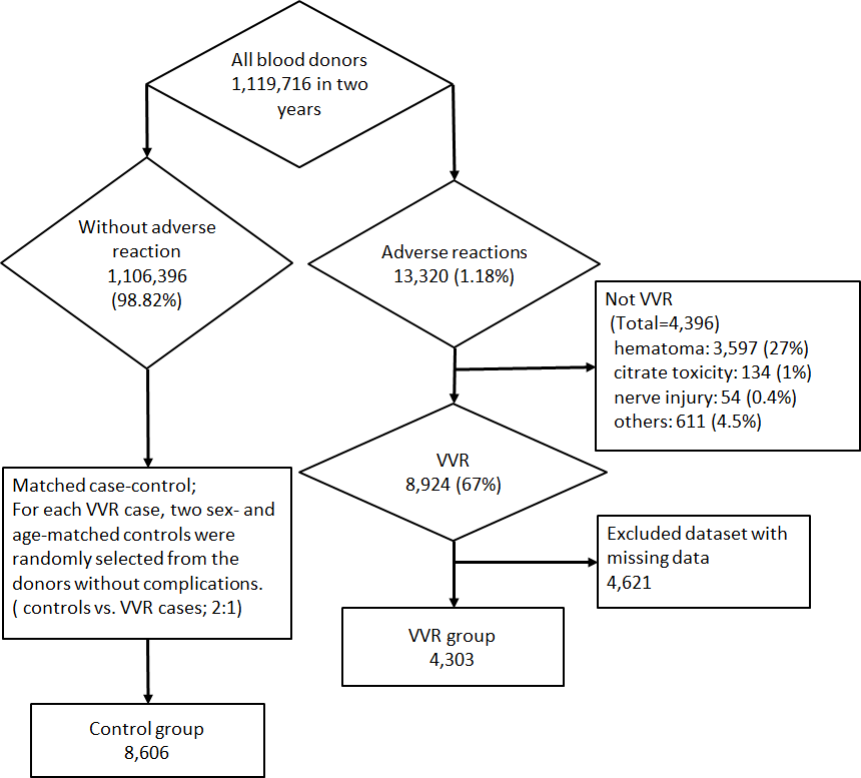
Flowchart of the study population.

The data from 1,119,716 donations collected at the JRC Tokyo Blood Center for two years, from April 1, 2006 to March 31, 2008, were extracted from the central database and subjected to unlinkable anonymization to be used in the present study. The donor data comprised 13,320 cases (1.18%) of adverse reactions, of which 67% were VVR, 27% hematoma, 1% citrate toxicity and 0.4% nerve injury. Only 400WB and PC & PPP donations were adopted for the present study. The dataset included the times of occurrence and recovery, and the blood volume lost, together with the donor’s height, weight, blood pressure, pulse rate, donation status and blood test results (TP, Alb and Hb). Excluding any dataset with missing data, there were 4,303 eligible cases enrolled in this study, of which 65 cases included fainting. The flowchart of the study population is shown in Fig 1.

This study was approved by the Research Ethics Review Committee of the Faculty of Sports and Health Science, Daito Bunka University (registration number: 09–029).

### Control group

The control group comprised of 8,606 age- and sex-matched cases randomly extracted from among subjects without adverse reactions to blood collection at a numerical ratio of 2:1 (controls vs. VVR cases) (Fig 1). We set the conditions according to the ‘Strengthening the reporting of observational studies in epidemiology’ (STROBE) recommendations [13].

### Statistical analysis

All donor information from the JRC Blood Centers was anonymized and de-identified prior to analysis at Daito Bunka University. Using descriptive statistical analyses, age, sex, height, weight, BMI, CBV, predonation systolic blood pressure (SBP), predonation diastolic blood pressure (DBP), predonation pulse rate, donation status, donation type, TP, Alb and Hb were analyzed as subgroups for evaluating the baseline characteristics of blood donors. For the VVR and control groups, the unpaired t-test was used in the univariate analysis for continuous numerical variables, while the chi-square test was used for categorical variables separated into two groups. The Cochran-Armitage trend test was applied to TP, Alb and Hb after dividing the values into quartiles. For multivariate analysis, conditional logistic regression was used; with the dependent variable being the presence or absence of VVR occurrence, and the independent variables being divided at each median value. Confounding factors including sex, BMI, SBP, pulse rate and donation status were used for adjustment to calculate the odds ratio (OR) and 95 percent confidence interval (95%CI). Furthermore, the unpaired t-test and the same-subgroup multivariate analysis using BMI, SBP, pulse rate and donation status as independent variables were conducted after stratification by sex to confirm the presence or absence of any difference between the sexes. All statistical analyses were performed using SAS statistical analysis software (ver. 9.1.3, SAS Institute Inc., Cary, NC) [14].

## Results

### Donor characteristics and multivariate analysis

Tables 1 and 2 show the characteristics of the VVR and control groups. The height, weight, BMI and CBV were lower in the VVR group than in the control. Predonation blood pressure, predonation pulse rate and levels of TP, Alb and Hb were higher in the VVR group than in the control group.

**Table 1:** Characteristics of the VVR and control groups (1).

Variable	VVR	Control	p_value
Overall	n = 4303	n = 8606	
	(mean±S.D.)	(mean±S.D.)	unpaired t-test
**Age (years)**	29±10.2	29±10.2	NS
**Height (cm)**	164.1±8.5	165.7±8.2	<0.001
**Weight (kg)**	58.9±9.3	61.5±10.6	<0.001
**BMI**	21.8±2.6	22.3±2.9	<0.001
**Circulating blood volume (L)**	4.1±0.6	4.2±0.7	<0.001
**Predonation systolic blood pressure (mmHg)**	117.2±14.2	116.6±14.7	0.0296
**Predonation diastolic blood pressure (mmHg)**	69.6±10.6	68.5±11.0	<0.001
**Predonation pulse rate (beats/min)**	77.9±12.1	76.3±11.6	<0.001
**Total protein (g/dL)**	7.3±0.4	7.1±0.4	<0.001
**Albumin (g/dL)**	4.7±0.2	4.6±0.2	<0.001
**Hemoglobin (g/dL)**	14.4±1.3	14.1±1.3	<0.001

**Table 2:** Characteristics of the VVR and control groups (2).

Variable	VVR	Control	chi-square test
	n (%)	n (%)	
**Sex**			NS
Female	2096(48.7)	4192(48.7)	
Male	2207(51.3)	4414(51.3)	
**Donation status**			<0.001
Repeat donor	2830(65.8)	7419(86.2)	
First-time donor	1473(34.2)	1187(13.8)	
**Donation type**			NS
PC & PPP	1637(38)	3357(39)	
400WB	2666(62)	5249(61)	

None of the values were determined to be abnormal. The chi-square test revealed a significantly higher ratio of first-time donors in the VVR group (p < 0.001), but no significant difference with respect to donation type. TP and Alb values showed no difference between males and females.

**Fig 2:**
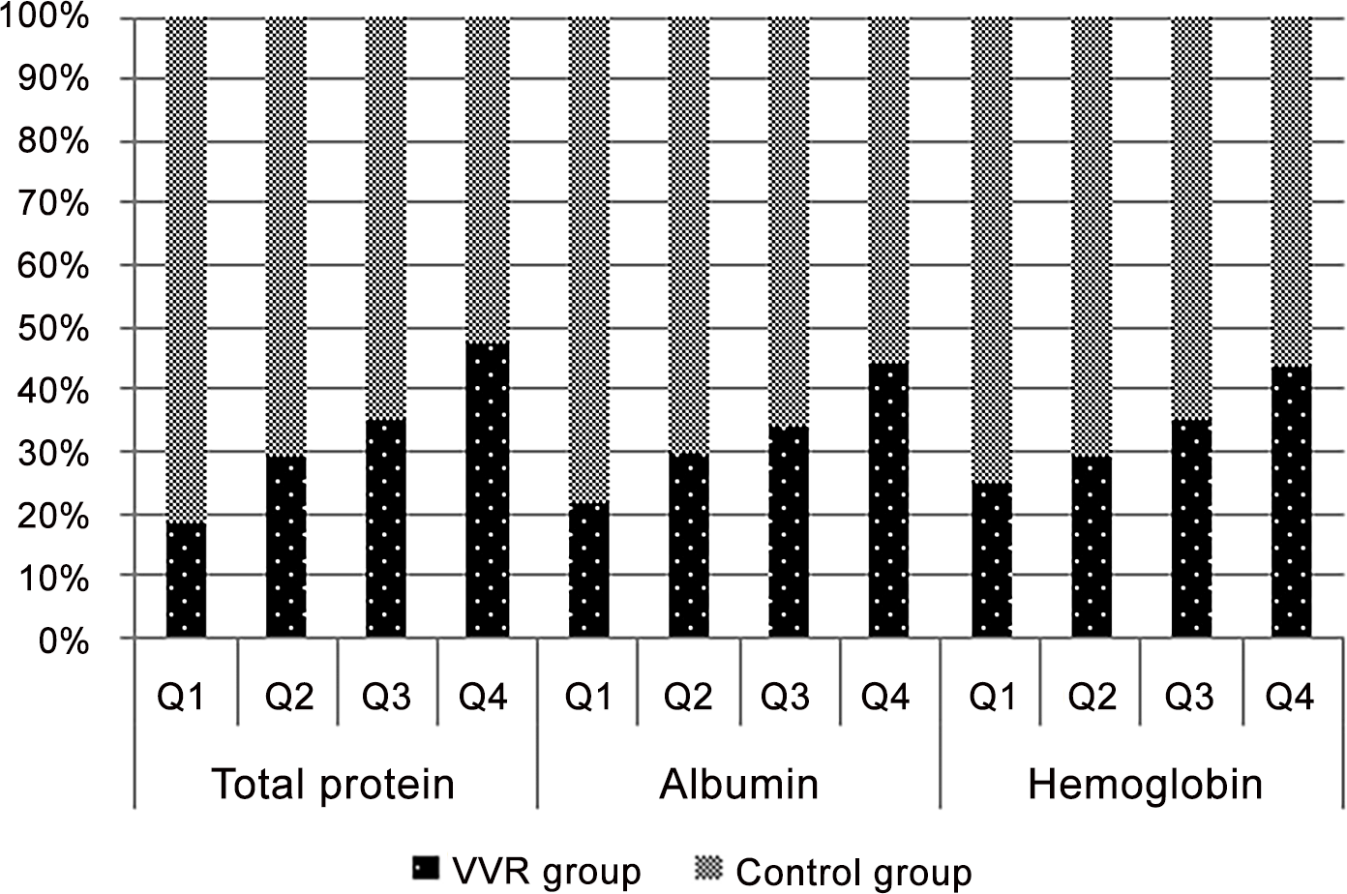
Trend test for the male subgroup (VVR-control ratio). The trend test was significant (p<0.001), and the quartiles were: Total protein (Q1,<6.9; Q2,<7.2; Q3,<7.5; Q4,≥7.5), Albumin (<4.5; <4.7; <4.9; ≥4.9), Hemoglobin (<14.5; <15.1; <15.7; ≥15.7).

**Fig 3:**
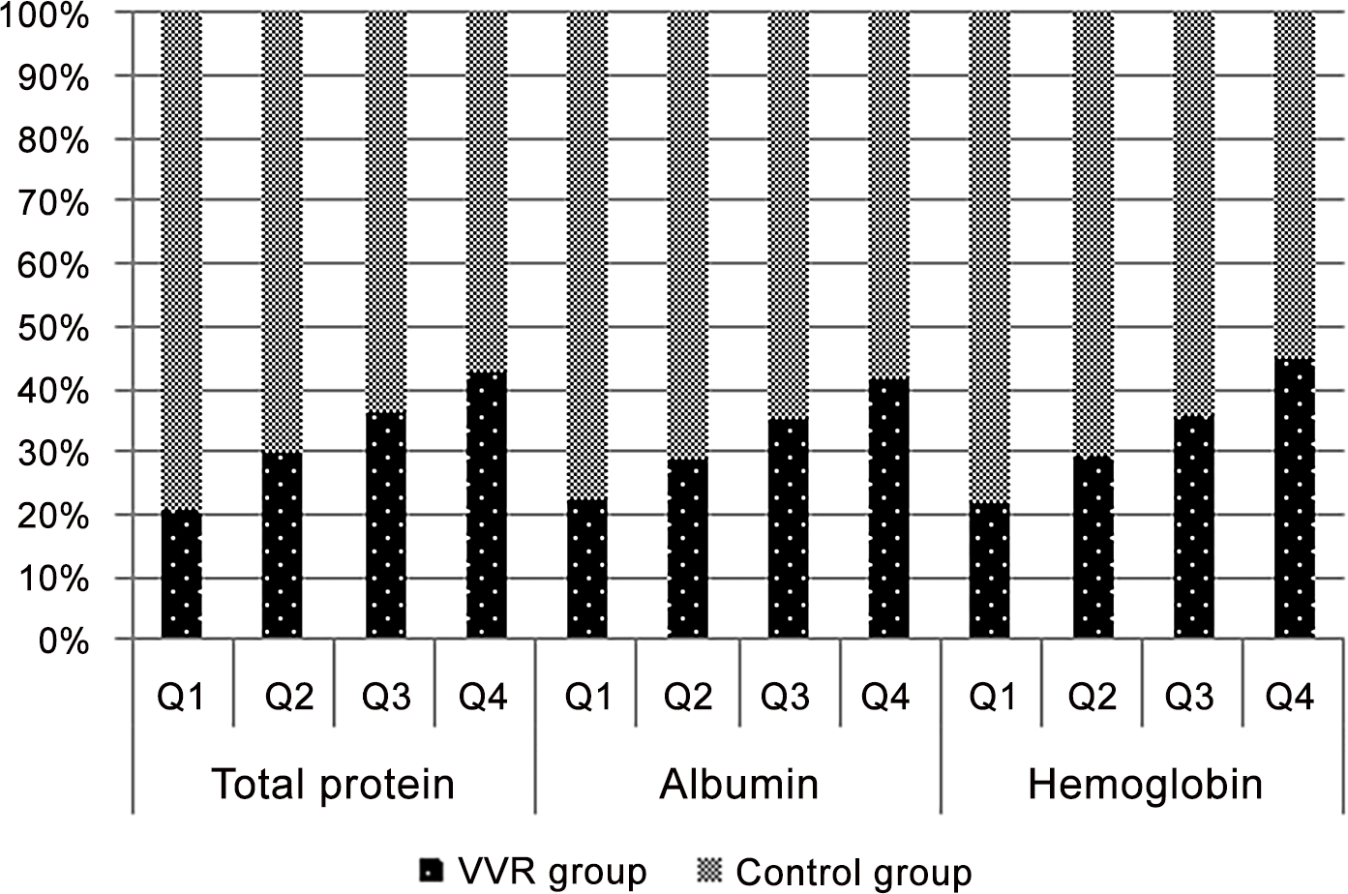
Trend test for the female subgroup (VVR-control ratio). The trend test was significant (p<0.001), and the quartiles were: Total protein (Q1,<6.9; Q2,<7.2; Q3,<7.5; Q4, ≥7.5), Albumin (<4.4; <4.6; <4.8; ≥4.8), Hemoglobin (<12.7; <13.2; <13.7; ≥13.7).

Figs 2 and 3 show the results of the Cochran-Armitage trend test in the sex subgroups. The trend test of these values revealed a significant difference (p < 0.001) and dose response in which the incidence of VVR increased as the values of TP, Alb and Hb increased.

Table 3 shows the results of the conditional logistic regression model. The adjusted OR(AdOR) of TP, Alb and Hb in the higher-value groups to those in the lower-value groups adjusted for confounding factors and their 95%CI were: TP (AdOR, 2.11; 95%CI, 1.95–2.29), Alb (1.70; 1.56–1.85) and Hb (1.84; 1.64–2.06), each showing a significantly increased risk.

**Table 3:** Multivariate analysis of VVR risk.

Demographic characteristic	VVR	Control	Total	Unadjusted OR	Adjusted OR*
	number (%)	number (%)	number (%)	(95% CI)	(95% CI)
Overall	n = 4303	n = 8606	n = 12909		
**Sex**					
Female	2096(33.3)	4192(66.7)	6288(48.7)	1(0.93–1.08)	1.12(1.03–1.22)
Male	2207(33.3)	4414(66.7)	6621(61.3)	1	1
**BMI**					
≥21.6	2016(30.4)	4614(69.6)	6630(51.4)	1	1
<21.6	2287(36.4)	3992(63.6)	6279(48.6)	1.33(1.23–1.43)	1.36(1.25–1.47)
**Circulating blood volume (L)**					
≥4.1	2006(29.7)	4751(70.3)	6757(52.3)	1	1
<4.1	2006(37.3)	3855(62.7)	6152(47.7)	1.42(1.32–1.53)	1.79(1.60–2.01)
**Predonation systolic pressure (mmHg)**					
≥115	2334(34.5)	4432(65.5)	6766(52.4)	1.12(1.04–1.21)	1.14(1.00–1.18)
<115	1969(32.1)	4174(67.9)	6143(47.6)	1	1
**Predonation diastolic pressure (mmHg)**					
≥68	2348(35.7)	4236(64.3)	6584(51)	1.27(1.17–1.37)	1.2(1.11–1.31)
<68	1955(30.9)	4370(69.1)	6325(49)	1	1
**Predonation pulse rate (beats/min)**					
≥76	2370(35.9)	4227(64.1)	6597(51.1)	1.27(1.18–1.37)	1.31(1.21–1.41)
<76	1933(30.6)	4379(69.4)	6312(48.9)	1	1
**Total protein (g/dL)**					
≥7.2	2815(40.6)	4125(59.4)	6940(53.8)	2.08(1.92–2.24)	2.11(1.95–2.29)
<7.2	1488(24.9)	4481(75.1)	5969(46.2)	1	1
**Albumin (g/dL)**					
≥4.6	3120(37.0)	5307(63.0)	8427(65.3)	1.69(1.56–1.84)	1.70(1.56–1.85)
<4.6	1183(26.4)	3299(73.6)	4482(34.7)	1	1
**Hemoglobin (g/dL)**					
≥14.1	2420(36.9)	4140(63.1)	6560(50.8)	1.39(1.29–1.50)	1.84(1.64–2.06)
<14.1	1883(29.7)	4466(70.3)	6349(49.2)	1	1
**Donation status**					
Repeat donor	2830(27.9)	7321(72.1)	10151(78.6)	1	1
First-time donor	1473(53.4)	1285(46.6)	2758(21.4)	3.79(3.45–4.17)	3.92(3.55–4.32)
**Donation type**					
PC & PPP	1637(32.8)	3357(67.2)	4994(38.7)	0.96(0.89–1.04)	1.29(1.18–1.40)
400WB	2666(33.7)	5249(66.3)	7915(61.3)	1	1

* The OR were adjusted for the sex, BMI group, systolic blood pressure group, pulse rate group and donation status group.

### Subgroup analysis by sex

For TP and Alb there was no difference between males and females, however the Hb level was higher in males than in females.

The male blood donors in the VVR group had significantly lower height (170.4 vs. 171.6 cm), weight (64.1 vs. 67.4 kg), BMI (22.1 vs. 22.9) and CBV (4.5 vs. 4.7 L) and significantly higher DBP (71.8 vs. 70.8 mmHg) (p < 0.01) than the control group. In addition, the males in the VVR group had significantly higher TP (7.3 vs. 7.2 g/dL), Alb (4.8 vs. 4.7 g/dL) and Hb (15.4 vs. 15.0 g/dL) (p < 0.001) than the control group. Female blood donors in the VVR group had significantly lower height (157.5 vs. 159.4 cm), weight (53.5 vs. 55.3 kg), BMI (21.6 vs. 21.8) and CBV (3.7 vs. 3.8 L) and a significantly higher pulse rate (79.5 vs. 76.7 beats/minute) and DBP (67.3 vs. 66.0 mmHg) (p < 0.01) than the control group. In addition, females in the VVR group had significantly higher TP (7.3 vs. 7.1 g/dL), Alb (4.7 vs. 4.6 g/dL) and Hb (13.4 vs. 13.1 g/dL) (p < 0.001) than the control group.

Table 4 shows the results of the conditional logistic regression after stratification by sex. The OR of TP, Alb and Hb in the higher-value groups to those in the lower-value groups adjusted for confounding factors and their 95%CI for males were: TP (1.97; 1.76–2.21), Alb (1.75; 1.55–1.96) and Hb (1.98; 1.76–2.22), each with a significantly increased risk. For females, they were: TP (2.29; 2.05–2.56), Alb (1.76; 1.57–1.97) and Hb (1.62; 1.45–1.81), each also with a significantly increased risk.

**Table 4:** Multivariate analysis of VVR risk in sex subgroups.

Demographic characteristic	Male				Female			
	VVR number (%)	Control number (%)	Unadjusted OR (95% CI)	Adjusted OR* (95% CI)	VVR number (%)	Control number (%)	Unadjusted OR (95% CI)	Adjusted OR* (95% CI)
Overall	n = 2207	n = 4414			n = 2096	n = 4192		
**Total protein (g/dL)**								
≥7.2	1428(39.4)	2194(60.6)	1.86(1.68–2.07)	1.97(1.76–2.21)	1387(41.8)	1931(58.2)	2.33(2.09–2.60)	2.29(2.05–2.56)
<7.2	779(26.0)	2220(74.0)	1	1	709(23.9)	2261(76.1)	1	1
Albumin (g/dL)^**^								
High	1466(38.2)	2374(61.8)	1.77(1.58–1.97)	1.75(1.55–1.96)	1340(38.7)	2122(61.3)	1.80(1.61–2.02)	1.76(1.57–1.97)
Low	741(26.6)	2040(73.4)	1	1	756(26.8)	2070(73.2)	1	1
Hemoglobin (g/dL)^***^								
High	1414(40.5)	2077(59.5)	2.03(1.82–2.25)	1.98(1.76–2.22)	1241(39.5)	1904(60.5)	1.75(1.57–1.95)	1.62(1.45–1.81)
Low	793(25.3)	2337(74.7)	1	1	855(27.2)	2288(72.8)	1	1
**Donation status**								
Repeat donor	1221(24.7)	3716(75.3)	1	1	1609(30.4)	3682(69.6)	1	1
First-time donor	986(58.6)	698(41.4)	5.94(5.20–6.79)	5.87(5.13–6.72)	487(48.8)	510(51.2)	2.34(2.03–2.71)	2.38(2.06–2.75)
**Donation type**								
PC & PPP	388(23.0)	1298(77.0)	0.51(0.44–0.57)	0.75(0.66–0.87)	1249(37.8)	2059(62.2)	1.54(1.39–1.72)	1.93(1.72–2.18)
400WB	1819(36.9)	3116(63.1)	1	1	847(28.4)	2133(71.6)	1	1

* The OR were adjusted for the BMI group, systolic blood pressure group, pulse rate group and donation status group.

^**^ The albumin groups were divided at the median: Males (High ≥4.7, Low <4.7), Females (High ≥4.6, Low <4.6).

^***^ The hemoglobin groups were divided at the median: Males (High ≥15.1, Low <15.1), Females (High ≥13.2, Low <13.2).

In the multivariate analysis, the male subgroup showed a higher risk for VVR when they had a BMI <22.2 (1.75; 1.56–1.97), CBV <4.5 (2.02; 1.80–2.26), SBP ≥121mmHg (1.20; 1.07–1.34) and DBP ≥70mmHg (1.21; 1.08–1.36). The female subgroup showed a higher risk for VVR when they had a BMI <21.2 (1.24; 1.11–1.38), CBV <3.7 (1.81; 1.62–2.02), DBP ≥65mmHg (1.18; 1.05–1.32) and pulse rate ≥77/min (1.57; 1.41–1.75). The donation type risk was shown to be different for males and females, with apheresis donation being a lower risk for males and a higher risk for females.

### Subgroup analysis by donation type

The higher value groups of TP, Alb and Hb, adjusted for confounding factors, showed a significantly increased risk compared to the lower value groups in the conditional logistic regression after stratification by donation type.

## Discussion

The VVR group had significantly lower values of height, weight, BMI and CBV and significantly higher values of predonation blood pressure and pulse rate than the control group. In addition, the incidence of VVR among first-time donors was higher compared to sex- and age-matched donors. Factors including lower BMI, higher predonation blood pressure and pulse rate, and being a first-time donor showed risks similar to those reported in many previous studies, including ours [4–10,15–17].

In this study, the VVR group had significantly higher TP, Alb and Hb values than the control group, although the values were in the normal range. In order to clarify the presence or absence of any difference between the sexes with respect to blood chemical values, we conducted the same subgroup analyses after stratifying the data by sex; the results were almost the same as those before the stratification. TP, Alb and Hb each showed a significantly higher risk in the higher value groups than the lower value groups even after adjusting for confounding factors. Furthermore, the trend tests revealed a dose response manner, indicating that VVR is more likely to occur as blood protein or Hb levels increase. The analysis with globulin (TP value minus Alb value) also showed a significant trend. The blood protein data can be a reflection of nutritional status, and a higher BMI is shown to be a lower risk for VVR. But in this study higher protein levels are associated with a higher VVR risk, indicating increased hemoconcentration rather than higher than usual levels of nutrition. A Brazilian study has shown that increasing hematocrit is closely associated with higher VVR risk [18]. Furthermore, high-school student donors given a drink of water had a 28 percent reduction in their VVR rate [19]. The study showed that the Hb level was lower 30 minutes after a drink.

Our previous studies of the JRC Blood Center’s donors have also indicated that water intake before and after blood collection reduces the occurrence of VVR [20,21]. A shift of interstitial fluid into intravascular space has been shown to occur during blood collection [22]. In our sex subgroup analysis (Table 4), apheresis donations have been shown to have different risks for males and females. Compared to 400WB donation, apheresis donation has a lower risk of VVR for males, but has a higher risk for females. The high VVR risk from apheresis donation for females has been reported in several studies [6,10,23,24]. The difference between the sexes might be associated with the blood volume and extravascular space. In our study, the CBV in the male and female control groups were 4.7 L and 3.8 L, respectively, and a CBV<4.1L was a risk factor for VVR. Therefore, the female population might be more vulnerable to VVR from apheresis donation. A lower BMI is reported to be a VVR risk factor [4,10], suggesting an association with the interstitial space.

Wiltbank et al. [4] reported that a systolic pressure >140 mmHg had a low VVR risk. They also reported a predonation pulse of <65 beats/minute is associated with a lower risk of VVR, and >90 beats/minutes with a higher risk of VVR. Ogata et al. [25] also reported that the lower the diastolic pressure, the higher the frequency of VVR, and stated that there was a positive correlation between age and blood pressure in WB200 donors. Our results showed that higher diastolic blood pressure and a higher pulse rate were each associated with a higher risk of VVR in WB400 and apheresis donors. The difference between our results and those in Ogata’s report may be due to the donation type studied (WB400 and apheresis vs. WB200), as well as the statistical methods and the cut-off value for the DBP, which was 68mmHg in this report. For blood pressure and pulse rate data, we should consider it not only to reflect stress, but also the possibility of dehydration with compensating cardiovascular function. In this report we have limitations in the details of the data for time and the amount of water intake, and do not have data for seasonal or site effects.

In the present study, a higher amount of hemoglobin or protein (hemoconcentration) was indicated to be a risk factor for VVR, as the incidence of VVR increased as the hemoconcentration status worsened. The biochemical data of the donors were within normal Japanese references. An important point is that the donors are healthy individuals of normal cardiovascular and renal function. They can normally deal with a slight negative balance of water, but not always when there is blood loss as well. It is possible that a negative balance of water results in a low volume of interstitial fluid, which is then insufficient to support the loss of intravascular volume during blood donation. There are reports that put emphasis on blood volume reduction in their pathogenesis [19–21], using the term VVR casually. The cardiovascular response and vasovagal reflex have been analyzed together. Timing data might help to understand the pathogenesis of VVR.

Because the present study was a cross-sectional design, a prospective interventional study would confirm the results. Including the body weight together with the percentage of body fat might help to calculate the reservoir space of body fluid. Our results suggest that checking the hemoconcentration status of a donor, such as with predonation testing, could give an opportunity to prevent VVR. As the Hb testing in donation rooms in Japan is by an automated blood cell counter, with venous puncture, intervention according to the Hb level is plausible.
